# Oral Manifestations of Systemic Lupus Erythematosus: A Systematic Review

**DOI:** 10.3390/ijerph191911910

**Published:** 2022-09-21

**Authors:** Paula García-Ríos, María Pilar Pecci-Lloret, Ricardo Elías Oñate-Sánchez

**Affiliations:** Gerodontology and Special Care Dentistry Unit, Morales Meseguer Hospital, Faculty of Medicine, IMIB-Arrixaca, University of Murcia, 30008 Murcia, Spain

**Keywords:** systemic lupus erythematosus, oral manifestations, oral ulcers

## Abstract

Systemic lupus erythematosus (SLE) is a chronic autoimmune disease that is characterized by clinical heterogeneity and irregularities in its course. The etiology and pathogenesis of this pathology are not well-understood, so there is difficulty in establishing a diagnosis and treatment plan with certainty. The aim of this systematic review is to present a qualitative synthesis of studies referring to the oral manifestations of systemic lupus erythematosus (SLE). This systematic review was performed following the PRISMA guideline. On this basis, a search for articles was performed in the PubMed, Web of Science, and Scopus databases on 19 November 2021 and updated on 15 February 2022. We chose articles published between 2012 and 2022 that analyzed the oral manifestations of SLE patients. The quality of all these studies was analyzed following the STROBE scale. A total of 15 articles were included in this study after selection. The selected articles were cross-sectional, case–control, and cohort studies. The most frequently associated oral manifestations with SLE were oral ulcers, hyposalivation, pigmentations, glossodynia, cleft tongue, cheilitis, arthritis, and secondary Sjögren’s syndrome. However, despite the importance of the perception of these oral manifestations in the early diagnosis of SLE, there are still not enough studies about them.

## 1. Introduction

Systemic lupus erythematosus (SLE) is defined as a chronic autoimmune disease that is characterized by heterogeneity in clinical presentation and systemic involvement [1,2]. Nuclear and cytoplasmic antigens are attacked by the produced antibodies [3], which has systemic repercussions. Although it can affect any organ, it most commonly affects the joints, skin, lungs, heart, nervous system, blood vessels, and liver [3,4]. The complexity in the management of this disease lies not only in the wide range of manifestations that it can present, but also in the irregularities of its course. This is because there are periods of crisis alternating with the remission of symptoms [3].

SLE’s actual prevalence and distribution are not well-known [3]. Several studies claim that ethnicity may influence SLE’s clinical presentation, complexity, and incidence. Thus, African-American and Hispanic individuals are more susceptible to SLE [3,5]. The autoimmune disease in question often appears in the late second and early third decades [5], and affects 90% of the female population of childbearing age [6]. On the other hand, the genetic component seems to influence susceptibility to SLE, as patients with relatives with SLE have a slightly higher risk of developing the disease [3].

Its etiology is unknown, but some pathophysiological mechanisms that may be involved in triggering SLE are evident. These include genetic, epigenetic, and environmental factors [3,7].

SLE is a highly variable clinical entity with multiple organ involvement [1,3]. The diagnosis of SLE is based on an expert assessment of clinical manifestations and classification criteria. The latter was updated in September 2019 by the European League Against Rheumatism (EULAR) and the American College of Rheumatology (ACR) [8]. On this basis, signs and symptoms were divided into two groups: those affecting the patient’s general constitution, and those affecting the different organs, apparatuses, and systems of the person with SLE [3,9].

Regarding constitutional signs and symptoms, fatigue (the most common symptom, although this is due to the coexisting factors of this autoimmune disease rather than the disease activity itself), fever, and weight loss before diagnosis are prominent [5]. It is essential to know the oral manifestations, as they are among the first to appear and they help in conducting an early diagnosis. Oral signs and symptoms were grouped according to the cutaneous LE group. Thus, chronic cutaneous SLE is characterized by well-demarcated, red, round or irregular, atrophic or ulcerated oral lesions that are asymmetrically distributed [10]. Subacute cutaneous SLE has red and round eruptions, although oral lesions are rare. In acute cutaneous SLE, there are mainly ulcers and blisters. Other orofacial manifestations include candidiasis, dysphagia, and xerostomia. Lastly, as it has a similar clinical presentation at the oral level to that of other entities such as lichen planus, pemphigus, or syphilis, a differential diagnosis must be conducted between SLE and these pathologies [1,3,11].

Knowledge of these signs and symptoms in the oral cavity favors early diagnosis, which improves the patient’s prognosis and quality of life. Hence, summarizing studies that provide information about the most frequent oral manifestations in patients with SLE is important. Thus, this systematic review presents a qualitative synthesis of studies referring to the oral manifestations of systemic lupus erythematosus (SLE).

## 2. Materials and Methods

### 2.1. Declaration and Protocol

This systematic review was conducted following the Preferred Reporting Items for Systematic reviews and Meta-Analyses (PRISMA) guidelines [12]. In addition, the systematic review was registered in PROSPERO (CRD42021291356).

### 2.2. Inclusion and Exclusion Criteria

Articles published between January 2012 and February 2022, those identifying and analyzing oral manifestations in SLE patients, and those that matched our search terms were included in our review; only English and Spanish studies were included. On the other hand, articles that reported only systemic manifestations of this pathology and thus did not meet the inclusion criteria were excluded.

To establish the inclusion criteria, they had to follow the PCO model: population/problem (P): patients with systemic lupus erythematosus; comparison/control (C): healthy patients; outcome (O): oral manifestations present in SLE patients. Thus, our PICO question is: which are the oral manifestations in patients with systemic lupus erythematosus?

### 2.3. Search Strategy

#### 2.3.1. Sources of Information

To identify and analyze the articles that incorporated relevant information to the proposed topic of this systematic review, an exhaustive search was carried out in the following databases: PubMed, Web of Science, and Scopus. This search was conducted on 19 November 2021 and updated on 15 February 2022.

#### 2.3.2. Search Terms

The terms used for the search were obtained from the Medical Subject Heading (Mesh) thesaurus. Those referring to the term “systemic lupus erythematosus” are as follows: “systemic lupus erythematosus”, “lupus erythematosus disseminates”, “libman-sacks disease”, “disease, libman-sacks”, “libman sacks disease”. Those referring to the term “oral manifestations” are as follows: “oral manifestations”, “manifestation, oral”, “manifestations, oral”, “oral manifestation”. Boolean operators (“AND” and “OR”) were used to relate the above terms to each other. The following table shows the obtained results from the search in the different databases (Table 1).

### 2.4. Study Selection

The studies resulting from the search process were entered into bibliographic manager Mendeley (Elsevier) to discard duplicates. Subsequently, the first selection of articles was carried out taking into account their title and abstract, and in compliance with the previously established inclusion and exclusion criteria. Lastly, the full text of the selected studies was read and analyzed to confirm their eligibility.

### 2.5. Data Extraction

Data extraction was performed with PGR. The following differentiated categories were taken into account for each of these articles: authorship and year of publication, type of study, most frequent manifestations, most frequent locations in the oral cavity, treatment, differential diagnosis, and associated conditions.

### 2.6. Quality Analysis

The quality of the studies included in this systematic review was analyzed by consensus among all authors using the Strengthening the Reporting of Observational Studies in Epidemiology (STROBE) scale [13], which established a series of recommendations on what an observational study should include. The STROBE scale consists of a list of 22 items. Each criterion was scored as positive with a tick (✔) when the requirement had been met, and as negative with a cross (🗶) when the requirement had not been met. All 22 criteria were selected, and studies with 16–22 points were considered of having a low risk of bias, 8–15 were considered to have a moderate risk, and those with 7 or less had a high risk of bias. The final study ratings for each assessor were collated and examined for discrepancies. Any disagreement between assessors was resolved by a consensus decision.

## 3. Results

**Figure 1:**
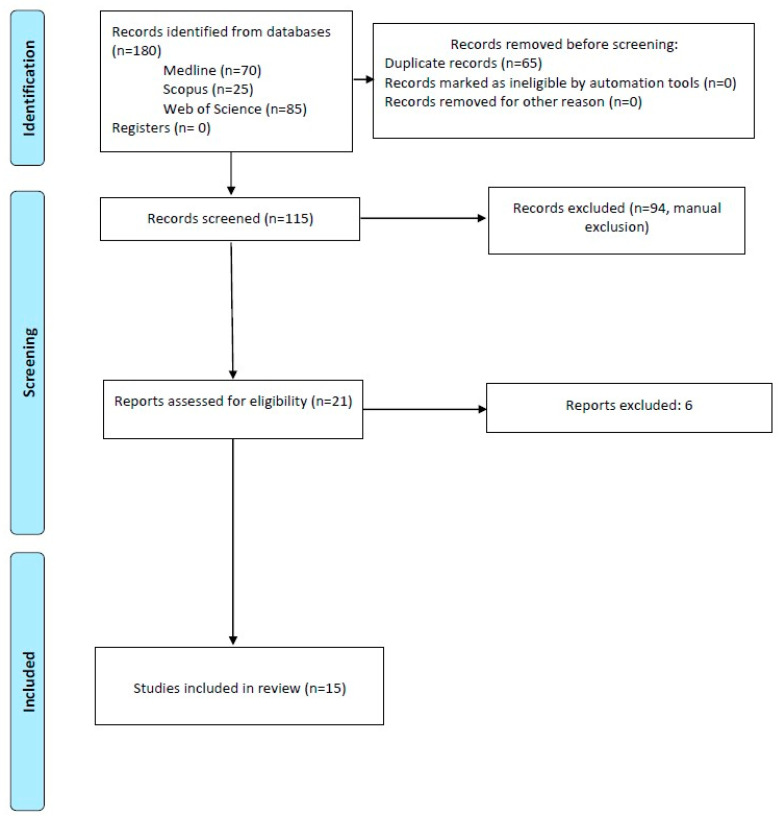
Systematic flow diagram.

The results of the study selection are shown in Figure 1. A total of 180 references were identified through an exhaustive database search, of which 70 belonged to Medline Pubmed, 25 to Web of Science, and 85 to SCOPUS. Subsequently, using bibliographic manager Mendeley, 65 duplicate articles were discarded, and the title and abstract of 115 references were analyzed. After examining them, 94 articles were excluded, so only 21 were read in full text, discarding 6 and lastly obtaining 15.

### 3.1. Results of Data Extraction

The results of the data extraction are represented in Table 2 and Table 3, showing the different categories mentioned above and the significance of the association of oral manifestations as part of the SLE clinic. This was established via *p*-value analysis.

**Figure 2:**
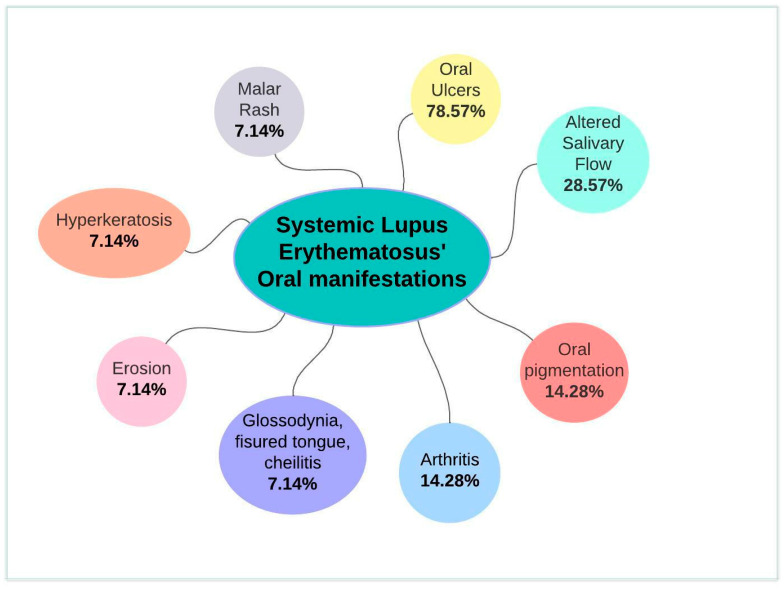
Summary of systematic lupus erythematosus’ oral manifestations.

Of the 14 studies that evaluated the oral manifestation prevalence of SLE, 11 concluded that it was oral ulcers (Table 2); a summary of the SLE’s oral manifestations can be found on Figure 2. Six articles evaluated the more common localization, and all agreed on hard palate (Table 2).

### 3.2. Results of Quality Analysis

The results of quality analysis are referenced in Table 4.

Concerning the bias of the published articles, a higher prevalence of low risk of bias studies was observed, with 11 of the 15 articles included in this review. The remaining 4 were of moderate risk of bias, with none with high risk of bias.

### 3.3. Bibliometric Analysis

**Figure 3:**
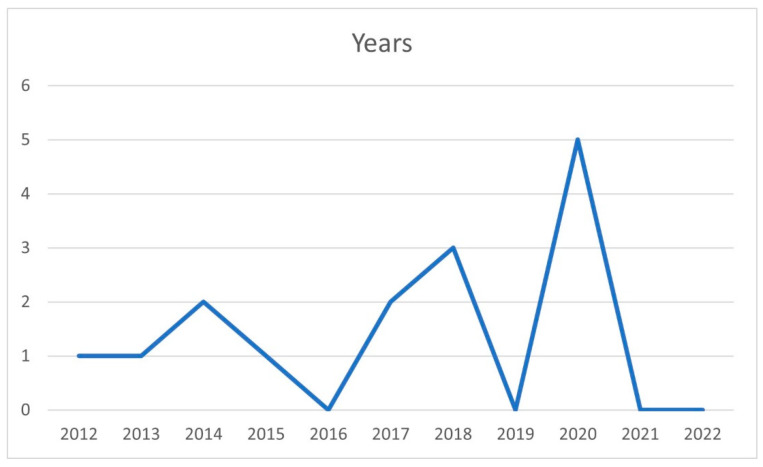
Distribution by year of publication.

**Figure 4:**
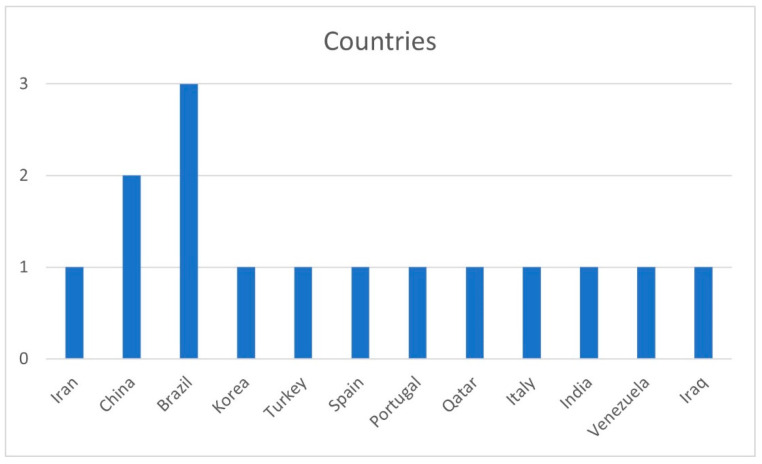
Distribution by country of publication.

**Figure 5:**
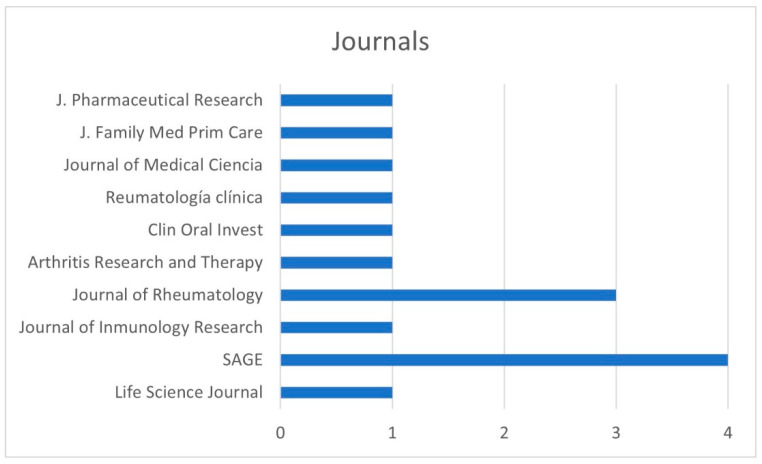
Distribution by journal of publication.

The distribution of the articles was by year of publication (Figure 3), country (Figure 4), and journal (Figure 5).

With regard to the year of publication (Figure 3), there was an increase in the number of articles published over time, which is beneficial for research. No studies were published in 2016, 2019, 2021, and 2022, while a total of 5 articles were published in 2020.

With regard to the country of publication (Figure 4), there was a higher prevalence of studies published in Brazil, where we found 3 articles, followed by China, where 2 articles were published. In the rest of the countries, there was 1 publication per country.

Numerous journals included articles related to SLE and its oral manifestations (Figure 5); among them was SAGE, where 4 studies were published, and the Journal of Rheumatology which included 3. 

## 4. Discussion

Systemic lupus erythematosus is an autoimmune disease characterized by its clinical heterogeneity, and a lack of knowledge about its etiology and prevalence [1]. This complicates the diagnosis and treatment plan.

Many studies have been carried out on this pathology to establish a series of aspects that facilitate early diagnosis and thereby an early approach to avoid severe complications. These studies have shown that the oral manifestations of SLE are among the first signs and symptoms to appear, hence the importance of understanding them to facilitate the management of this disease [3]. Therefore, the aim of this systematic review was to group and analyze those studies that referred to the oral manifestations of SLE, emphasizing the most frequent ones and their possible associations.

According to Zakeri et al., oral involvement was observed in 61.4%, and the most prevalent were oral aphthous ulcers, erosion, hyperkeratosis and pigmentation, which occurred mainly on the hard palate, followed by the soft palate and the vermilion of the lower lip [14].

As previously mentioned, of all the manifestations that could appear at the oral level, the most prevalent is oral ulceration; in fact, it is one of the criteria to be considered when trying to classify SLE [8]. In addition, the early detection of ulcers is important because it allows for faster diagnosis and treatment, since failure to do so is associated with increased disease activity and a worse prognosis [16]. Other diseases also rely on the occurrence of ulcers for their diagnosis, such as COVID-19, lichen planus, pemphigus, or syphilis, so a differential diagnosis should be performed [11,29,30,31,32].

Ali et al. reported that there was a possible association between the polymorphism of differentiation group 34 (which is an antigen found in immune cells) with oral ulceration in Iraqi patients with SLE, because CD34 polymorphisms may affect the immune system by triggering the development of oral ulcers [15].

On the other hand, Aterido et al. [16] studied the association between the vascular endothelial growth factor (VEGF) pathway with oral ulceration in SLE. They concluded that the VEGF pathway is responsible for regulating angiogenesis, so that the dysregulation of the VEGF pathway leads to ulcers. Not only was the polymorphism of differentiation group 34 or the VEGF pathway associated with the occurrence of oral ulcers in SLE patients, but the presence of the APL antibody in these patients was also significantly associated with a lower prevalence of oral ulcers [17].

Although the most prevalent oral manifestation is the appearance of oral ulcers, we could also find other conditions such as hyposalivation, hyperpigmentation due to antimalarials, and the presence of lesions in the oral mucosa, dental caries, and periodontal disease. Leite et al. [18] studied the prevalence of hyposalivation and stated that more than 75% of SLE patients suffer from hyposalivation (decreased salivary flow), which could also lead to the development of xerostomia (dry mouth sensation); with increasing age and SLE activity, the amount of saliva produced by the salivary glands decreases.

Similarly, in the case–control study conducted by Manzano et al. [19] a significant association was found among xerostomia, and lower unstimulated and stimulated total salivary flow volume and SLE patients. This influenced a poorer quality of life in these individuals with a greater effect on mental health.

Aurlene et al. [21] established a statistically significant association between patient age and the presence of gingival bleeding, and between disease activity and the prevalence of decayed and missing teeth. Periodontal involvement may be explained by the action of immunosuppressive drugs administered to this type of patient, which influences the growth of periodontal pathogens by inhibiting immune function. The presence of caries, on the other hand, develops due to decreased salivary flow rate leading to an imbalance in the bacterial flora. The increased prevalence of oral mucosal lesions is caused by the action of circulating antigen–antibody complexes leading to the degeneration of oral mucosal keratinocytes.

Hammoudeh et al. [22] agreed with these findings and also demonstrated the relationship among SLE, periodontal disease, and the presence of carious teeth. Periodontal disease was more common in patients with SLE with a duration of more than 8 years, but no statistically significant differences in gingivitis were found for this characteristic. This study also reported a higher prevalence of candidiasis and infections in SLE patients due to the administered medication and established that the most frequent manifestation is the presence of oral ulcers.

Lastly, the publication by Crincoli et al. [23] also found a statistically significant association between hyposalivation and SLE. This implies an increased risk of developing caries, oral ulcers, gingivitis, periodontal disease, fissured tongue, fungal infections (especially candidiasis), angular cheilitis and glossodynia. In particular, oral ulcers, glossodynia, fissured tongue, and cheilitis were significantly associated with SLE patients. In addition, a higher prevalence of dysphagia, dysgeusia, and glossodynia was found. 

There are also studies comparing the clinical presentation in patients with juvenile-onset and adult-onset SLE [24,25,26,27,28]. A 15–20% of SLE patients develop the disease before adulthood, 2–20% after the age of 50, and the remainder in adulthood [25]. It appears that the age of disease onset has a significant relationship with disease expression and outcome [26]. In general, juvenile-onset SLE has a more aggressive course and organ involvement than adult- or late-onset SLE does, with a greater need for immunosuppressive medication for a longer period of time [24].

With regard to the oral manifestations present in both entities, a significant association was established in terms of a higher prevalence of oral ulceration in patients with juvenile-onset SLE compared to those who develop the disease at the beginning or during adulthood [24,25,26]. Fonseca et al. [24] determined that oral ulceration was 45.5% prevalent in the young group compared to 17.5% in adults. Malar rash was also more prevalent in the young group, but arthritis that can affect the TMJ and Sjögren’s syndrome were established as the predominant conditions in adult patients [24]. Choi et al. [25] divided SLE into three groups: Juvenile SLE (up to 18 years), adult SLE (19 to 50 years), and late SLE (over 50 years). Fonseca et al. [24] reported that oral ulcers were significantly more frequent in people with juvenile-onset SLE than in those patients who were adults or older than 50 years. Sjögren’s syndrome was significantly more prevalent in patients with late SLE [25]. Artim-Esen et al. [26] agreed that oral ulceration was significantly higher in the juvenile group (23.1%) than in adult patients 15.4%. Novak et al. [28] established that oral ulcers were significantly more frequent in patients and when the time to a diagnosis was more than three months, they could be associated with the treatment. 

This review and the other published articles have limitations. Among them, we found a small number of different articles that provided sufficient information on oral (nonsystemic) manifestations in the used databases, as most of them were repetitive. 

## 5. Conclusions

The most frequently associated oral manifestations with SLE are oral ulcers, hyposalivation, pigmentations, glossodynia, cleft tongue, cheilitis, arthritis, and secondary Sjögren’s syndrome. Furthermore, the presence of these signs and symptoms in the oral cavity is frequent, hence the importance of their study. The high prevalence of oral ulcers, especially in young patients, and hyposalivation in patients with adult- or late-onset SLE is noteworthy. However, despite the importance of the perception of these oral manifestations in the early diagnosis of SLE, because they are usually among the first clinical manifestations of this pathology to appear, there are still not enough studies about them.

## Figures and Tables

**Table 1:** Search strategy.

Base of Data	Search Field	Results
Medline (PubMed)	(1) “Systemic lupus erythematosus” OR “lupus erythematosus disseminates” OR “Libman–Sacks disease” OR “disease, Libman–Sacks” OR “Libman–Sacks disease”.	73,780
	(2) “Oral manifestations” OR “manifestation, oral”. OR “manifestations, oral” OR “oral manifestation”.	4311
	**1 AND 2**	**71**
Web of Science	(1) “Systemic lupus erythematosus” OR “lupus erythematosus disseminatus” OR “Libman–Sacks disease” OR “disease, Libman–Sacks” OR “Libman–Sacks disease”.	75,149
	(2) “Oral manifestations” OR “manifestation, oral” OR “manifestation, oral”. OR “manifestations, oral” OR “oral manifestation”.	1826
	**1 AND 2**	**25**
SCOPUS	(1) “Systemic lupus erythematosus” OR “lupus erythematosus disseminatus” OR “Libman–Sacks disease” OR “disease, Libman–Sacks” OR “Libman–Sacks disease”.	95,177
	(2) “Oral manifestations” OR “manifestation, oral” OR “manifestation, oral”. OR “manifestations, oral” OR “oral manifestation”.	4937
	**1 AND 2**	**85**

**Table 2:** Description of the differentiated variables.

Author	Year	Type of Study	Most Prevalent Manifestations	Most Frequent Locations	Associated Conditions
Zakeri et al. [14]	2012	Cross-sectional	Erosion, hyperkeratosis, oral pigmentation, and oral ulcers	Hard palate, soft palate, and lower lip	-
Ali et al. [15]	2020	Case–control	Oral ulcers	-	Haplotype C of CD34 gene polymorphism associated with oral ulcers.
Aterido et al. [16]	2017	Cohort	Oral ulcers	-	VEGF pathway, increased SLE activity, and worse prognosis associated with oral ulcers.
Li et al. [17]	2014	Cohort	-	-	APL antibody and a lower prevalence of oral ulcers.
Leite et al. [18]	2015	Cross-sectional	More than 75% with dry mouth	-	Disease activity, medication, 27+ years associated with hyposalivation.
Manzano et al. [19]	2021	Case–control	Decreased salivary flow rate	-	Negative impact on quality of life and mental health related to xerostomia.
Chacon et al. [20]	2020	Cohort	Hyperpigmented macules due to medication	Hard palate, cheeks and tongue	-
Aurlene et al. [21]	2020	Cross-sectional	Oral ulcer	Hard palate	Patient age and gingival bleeding. Disease activity and decayed, missing teeth, gingival bleeding, increased probing depth, and oral mucosal lesions.
Hammoudeh et al. [22]	2018	Pilot study	Oral ulcers	Hard palate	Increased prevalence of candidiasis, infections, and periodontitis in SLE patients.
Crincoli et al. [23]	2020	Case–control	Oral ulcers, glossodynia, fissured tongue, cheilitis	Hard palate, buccal mucosa, and lips	Hyposalivation, TMJ, and muscle involvement with SLE.
Fonseca et al. [24]	2018	Cross-sectional	Oral ulceration, arthritis, and secondary Sjögren’s syndrome	-	Ulcers and juvenile SLE. Arthritis and Sjögren’s disease, and adult SLE.
Choi et al. [25]	2015	Cross-sectional	Oral ulcers and Sjögren’s syndrome	-	Ulcers and juvenile SLE. Sjögren’s disease and adult SLE.
Artim-Esen et al. [26]	2017	Cohort	Oral ulcers	-	Most common oral ulcers and viral infections in juvenile-onset SLE.
Lee et al. [27]	2013	Cohort	Malar rash, arthritis and oral ulceration	-	More common in pediatric-onset SLE.
Novak et al. [28]	2018	Cohort	Oral ulcers	Palate	Most frequent oral ulcers in patients with long interval to diagnosis.

**Table 3:** Oral signs and symptoms and their significant association.

Author	Parameter 1	Parameter 2	Significance Level
Zakeri et al. [14]	Prevalence of SLE	1. Age 2. Sex	1. Not significant, *p* = 0.3 2. Not significant, *p* = 0.35
Ali et al. [15]	CD34 gene haplotypes	1. Distribution A, D–H 2. Distribution B, C 3. Single or multiple ulcers 4. Pain associated with oral ulceration	1. Significant, *p* ≤ 0.001 2. Not significant, *p* = 0.22 and *p* = 0.21 3. Significant, *p* = 0.04 for E 4. Not significant, *p* ≥ 0.05
Aterido et al. [16]	Via VEGF	Presence of oral ulcers	Significant, *p* = 0.044
Li et al. [17]	1. APL antibody 2. Anti-Sm and anti-rRNP antibodies	1. Lower prevalence of oral ulcers 2. Malar rash	1. Significant, *p* < 0.05 2. Significant, *p* < 0.001 and *p* < 0.05
Leite et al. [18]	Severity of hyposalivation	1. SLE activity 2. Medication 3. Age (>27 years)	1. Significant, *p* = 0.004 2. Not significant, *p* = 0.442 3. Significant, *p* = 0.021
Manzano et al. [19]	LES	Lower salivary flow stimulated and not	Significant, *p* = 0.004 and *p* = 0.016
Chacon et al. [20]	Hyperpigmented macules	Antimalarials Retinal toxicity	1. Not significant 2. Not significant
Aurlene et al. [21]	1. Age 2. LES activity 3. LES activity	1. Gingival bleeding and decayed and missing teeth 2. Gingival bleeding, attachment loss, and oral mucosal injuries 3. Decayed teeth	1. Significant, *p* < 0.05 2. Significant, *p* < 0.001 3. Significant, *p* < 0.05
Hammoudeh et al. [22]	SLE > 8 years	Periodontitis	Significant, *p* = 0.002
Crincoli et al. [23]	LES	1. Headache in the temples and difficulty opening the mouth 2. Decreased salivary flow 3. Limited left protrusion and left laterality 4. Notches on lateral edges of the tongue 5. Cheilitis, fissured tongue and oral ulcers	1. Significant, *p* = 0.035 and *p* = 0.043 2. Significant, *p* < 0.0001 3. Significant, *p* < 0.001 and *p* = 0.0282 4. Significant, *p* = 0.007 5. Significant, *p* = 0.028, *p* = 0.006 and *p* = 0.045
Fonseca et al. [24]	Juvenile LES	Oral ulcers and malar rash	Significant, *p* = 0.001
	Adult SLE	Arthritis	Significant, *p* = 0.04
Choi et al. [25]	Juvenile LES	Oral ulcers	Significant, *p* = 0.022
	Late SLE	Sjögren’s syndrome	Significant, *p* = 0.021
Artim-Esen et al. [26]	Juvenile LES	Oral ulcers	Significant, *p* = 0.008
Lee et al. [27]	Sex	Oral ulcers	Not significant, *p* = 0.3152
Novak et al. [28]	SLE > 3 months after diagnosis	Oral ulcers	Significant, *p* = 0.032

**Table 4:** Quality analysis results.

	Zakeri et al. 2012 [14]	Ali et al. 2020 [15]	Aterido et al. 2017 [16]	Li et al. 2014 [17]	Leite et al. 2015 [18]	Manzano et al. 2021 [19]	Chacon et al. 2020 [20]	Aurlene et al. 2020 [21]	Hammoudeh et al. 2018 [22]	Crincoli et al. 2020 [23]	Fonseca et al. 2018 [24]	Choi et al. 2015 [25]	Artim-Esen et al. 2017 [26]	Lee et al. 2013 [27]	Novak et al. 2018 [28]
1	✔	✔	✔	✔	✔	✔	✔	✔	✔	✔	✔	✔	✔	✔	✔
2	✔	✔	✔	✔	✔	✔	✔	✔	✔	✔	✔	✔	✔	✔	🗶
3	✔	🗶	✔	🗶	✔	✔	✔	✔	✔	✔	✔	✔	✔	✔	✔
4	✔	✔	✔	🗶	✔	✔	🗶	🗶	🗶	✔	✔	🗶	✔	✔	✔
5	🗶	✔	✔	✔	✔	✔	✔	✔	✔	✔	✔	✔	✔	✔	✔
6	🗶	🗶	✔	✔	✔	✔	✔	🗶	🗶	✔	🗶	✔	✔	✔	✔
7	✔	✔	✔	✔	✔	✔	🗶	✔	✔	✔	✔	✔	✔	✔	✔
8	🗶	🗶	🗶	🗶	✔	✔	🗶	🗶	🗶	✔	🗶	🗶	🗶	✔	🗶
9	🗶	🗶	🗶	🗶	🗶	✔	🗶	✔	🗶	🗶	🗶	🗶	🗶	🗶	🗶
10	✔	🗶	✔	✔	✔	🗶	✔	✔	✔	✔	🗶	✔	✔	✔	✔
11	✔	✔	✔	✔	✔	✔	🗶	🗶	✔	✔	✔	✔	✔	✔	✔
12	✔	✔	✔	✔	✔	✔	✔	✔	✔	✔	✔	✔	✔	✔	✔
13	✔	🗶	✔	✔	✔	✔	✔	✔	✔	✔	✔	✔	✔	✔	✔
14	✔	🗶	✔	✔	✔	✔	✔	✔	✔	✔	✔	✔	✔	✔	✔
15	🗶	🗶	✔	✔	✔	✔	🗶	✔	✔	✔	✔	✔	✔	✔	✔
16	🗶	✔	🗶	✔	🗶	✔	🗶	✔	✔	🗶	✔	🗶	🗶	🗶	✔
17	✔	✔	🗶	✔	🗶	✔	✔	✔	✔	✔	✔	✔	✔	🗶	🗶
18	✔	🗶	✔	✔	✔	✔	✔	✔	✔	✔	✔	✔	✔	✔	🗶
19	✔	🗶	✔	✔	✔	✔	✔	✔	🗶	🗶	✔	✔	✔	✔	✔
20	✔	✔	✔	🗶	✔	🗶	🗶	✔	🗶	✔	✔	✔	🗶	✔	🗶
21	✔	🗶	✔	🗶	✔	✔	✔	✔	🗶	✔	✔	✔	🗶	✔	🗶
22	✔	🗶	✔	✔	🗶	✔	🗶	🗶	🗶	🗶	🗶	✔	🗶	🗶	✔
**Total** **Risk of Bias**	**16** **Low**	**10** **Mod**	**18** **Low**	**16** **Low**	**18** **Low**	**20** **Low**	**13** **Mod**	**17** **Low**	**14** **Mod**	**18** **Low**	**17** **Low**	**18** **Low**	**16** **Low**	**18** **Low**	**15** **Mod**

* Mod: Moderate.
